# Effects of Acetone Vapor on the Exciton Band Photoluminescence Emission from Single- and Few-Layer WS_2_ on Template-Stripped Gold

**DOI:** 10.3390/s19081913

**Published:** 2019-04-23

**Authors:** Samantha Matthews, Chuan Zhao, Hao Zeng, Frank V. Bright

**Affiliations:** 1 Department of Chemistry, Natural Sciences Complex, University at Buffalo, The State University of New York, Buffalo, NY 14260-3000, USA; smatthew@buffalo.edu; 2 Department of Physics, Fronczak Hall, University at Buffalo, The State University of New York, Buffalo, NY 14260-3000, USA; chuanzha@buffalo.edu (C.Z.); haozeng@buffalo.edu (H.Z.)

**Keywords:** single- and few-layer WS_2_, atomic force microscopy, Raman and Photoluminescence mapping, vapor sensing

## Abstract

Two-dimensional (2D) materials are being used widely for chemical sensing applications due to their large surface-to-volume ratio and photoluminescence (PL) emission and emission exciton band tunability. To better understand how the analyte affects the PL response for a model 2D platform, we used atomic force microscopy (AFM) and co-localized photoluminescence (PL) and Raman mapping to characterize tungsten disulfide (WS_2_) flakes on template-stripped gold (TSG) under acetone challenge. We determined the PL-based response from single- and few-layer WS_2_ arises from three excitons (neutral, A^0^; biexciton, AA; and the trion, A^−^). The A^0^ exciton PL emission is the most strongly quenched by acetone whereas the A^−^ PL emission exhibits an enhancement. We find the PL behavior is also WS_2_ layer number dependent.

## 1. Introduction

Transition metal dichalcogenides (TMDs), such as MX_2_, where M = Mo or W (but can be most transition metals from groups IV, V, VI, and VIII), and X = Se, S, or Te, consist of one metal atomic layer hexagonally packed between two chalcogenide atomic layers [1,2,3,4,5,6]. The metal possesses six-fold in-plane coordination and the chalcogenide layers can be trigonal or octahedral [1,2]. In the trigonal prismatic (2H) form, the TMD will be semiconducting; and in the octahedral form (1T), the TMD is metallic [3,6]. In either phase, the bond between the metal and chalcogen atom is covalent, and the layers are held together by weak van der Waals forces [4].

Transition metal dichalcogenides have been studied for many years because the bulk materials exhibit an indirect bandgap whereas their single-layer counterparts possess a direct bandgap [1,2,3,4,5]. Early thin layer TMD research focused on creating new, two-dimensional (2D) field-effect transistors (FETs), nanoelectronics, photo detectors, and sensors [7,8,9,10,11,12]. For example, conductance-/resistance-based measurements have been performed on single- and few-layer MoS_2_ to detect NO_2_, NH_3_, humidity, trimethylamine, acetone, and dichloromethane [9,10]. Resistance-based measurements using WS_2_ thin films at 150 °C, exhibit sensitivity to H_2_, NH_3_, and NO_2_ [12]. Similarly, Huo et al. [11] reported O_2_, air, EtOH, and NH_3_ sensing by using single- and few-layer WS_2_ as photo detectors. Theoretical first-principles studies [13,14] have suggested that adsorbed NO, O_2_, H_2_O, and CO molecules on single- and few-layer WS_2_ can change the WS_2_ electronic and optical properties, making WS_2_ an attractive candidate for room temperature gas sensing platforms. In addition, Cho et al. [15] used photoluminescence (PL)-based measurements on single- and few-layer MoS_2_ to detect NO_2_ and NH_3_. To the best of our knowledge, there has yet to be any report exploiting the PL emission from single- and few-layer WS_2_ for chemical sensing. We have chosen WS_2_ as our model TMD because the PL emission from single-layer WS_2_ is 20 to 40 times more intense in comparison to single-layer MoS_2_ [16], and, where the PL gradually decreases with increasing film thickness in WS_2_, there is a drastic decrease from single- to bi-layer MoS_2_ [17].

WS_2_ has three main Raman active phonon modes (2LA(M), ~350 cm^−1^; E^1^_2g_, ~355 cm^−1^; and A_1g_, ~418 cm^−1^) [2,4]. The difference in energy between the E^1^_2g_ and A_1g_ bands (*Δ*) and the 2LA/A_1g_ intensity ratio depends on the number of WS_2_ layers [2]. Single- and few-layer WS_2_ also exhibits a strong PL emission and the emission spectrum arises from three excitons [4,18], including the neutral exciton (A^0^, an electron and hole bound together), biexciton (AA, two neutral excitons bound weakly), and trion (A^−^, two electrons and one hole). Previous single point PL emission experiments [15] on single-layer MoS_2_ specimens have shown that the A^0^ and A^−^ exciton bands behave differently when different gaseous vapors pass over the 2D specimen. For example, NO_2_ (electron acceptor)/NH_3_ (electron donor) vapors cause the A^−^ band amplitude to increase/decrease whereas the A^0^ band amplitude to decrease/increase [15].

Although most research has studied WS_2_ on SiO_2_/Si substrates, we chose gold substrates because of their applications in tip-enhanced Raman scattering (TERS) spectroscopy to enhance the Raman and PL signal and allow improved spatial resolution and scanning Kelvin probe microscopy (SKPM), to study the specimen’s electronic properties.

In this paper, we determine the acetone-induced changes in the A^0^, AA, and A^−^ exciton band amplitude, peak energy, and energy distribution across individual WS_2_ flakes that consist of single- and few-layer regions by using co-localized, confocal Raman, and PL emission mapping experiments.

## 2. Materials and Methods

### 2.1. WS_2_ Materials and Supplies

The following were used: Acetone (Fisher Scientific, Hampton, NH, USA); Ar gas (Jackson Welding&Gas Products, Buffalo, NY, USA); N_2_ gas (Airgas, Randor, PA, USA); polymethyl methacrylate (PMMA) (MW 120K, Sigma Aldrich, St. Louis, MO, USA); sulfur (≥99.95% purity, Alfa Aesar, Haverhill, MA, USA); tetrahydrofuran (THF, HPLC grade (Pharmco-Aaper, Brookfield, USA)); WO_3_ (99.98% purity, Acros Organics, VWR, Randor, PA, USA); Au evaporation pellets (Kurt J. Lesker Co., Jefferson Hills, PA, USA); Epo-Tek 377 epoxy (Epoxy Technology, Billerica, MA, USA); p-type, boron-doped silicon wafers (<100>) (650 to 690 µm thickness and 1 to 10 Ω·cm resistivity, Alsil Supply Division Ymart, Palm Beach Gardens, FL, USA); and sapphire substrates (Hefei Keijing Materials Technology, Hefei, China). 

### 2.2. 2D WS_2_ Fabrication 

Ultrathin WO_3_ films were deposited by electron beam evaporation onto sapphire substrates by using an ATC Series UHV dual chamber deposition system. These films, along with S were placed in consecutive zones inside a quartz furnace tube. A simplified diagram depicting the fabrication scheme [19] can be found in the Supplementary Materials in Figure S3. Zone 1 contained ~4 to 8 mg of S and was placed 30 cm upstream from zone 2, which held the WO_3_ film. Both zones had independently controlled temperatures, and the temperature ramping profiles were used to control the S vapor pressure at the WS_2_ growth temperature. The carrier gas was N_2_ with a flow rate of 40 standard cubic centimeters per minute (sccm) at ambient pressure. The zone 1 source temperature (containing S) was increased to 190 °C at a rate of 6 °C/min, and heating of this zone was stopped after 5 min. The temperature in zone 2 was increased at the same time as zone 1 to 745 °C at a rate of 25 °C/min. The temperature was then kept at 745 °C for 5 min to allow WS_2_ growth. After the 5 min growth time, the heaters and N_2_ flow were turned off to obtain single layer WS_2_ with some few layer overgrowth. A proposed WS_2_ from the WO_3_ mechanism has been described elsewhere [19,20].

### 2.3. Template Stripped Gold (TSG) Fabrication 

Template stripped gold was created in several steps [21]. Initially, 125 nm of Au was deposited onto an Si wafer by e-beam evaporation (AXXIS electron-beam evaporator with glancing angle deposition (Kurt J. Lesker Co.). This Au-coated wafer was cut into roughly 1 cm × 1 cm squares by using a diamond tip cutter. A second Si wafer, without Au coating, was cut into roughly 1.5 cm × 1.5 cm squares by using a diamond tipped cutter. A drop of epoxy was placed on the Au-coated Si wafer and the uncoated Si wafer was placed on top of this, effectively sandwiching the Au. The sample was then cured in an oven at 150 °C for 1 h. After cooling, the epoxy “sandwich” was placed in a beaker of THF for 1 h to facilitate the separation between the Au and the Si wafer it was deposited on. The resulting substrate was an ultra-flat Au surface with an RMS (root mean square) roughness of ~0.7 nm.

### 2.4. WS_2_ Exfoliation onto TSG 

WS_2_ samples were exfoliated onto TSG films by using the “water droplet method” [22]. Briefly, PMMA (1.2 M) was dissolved in chlorobenzene and spin coated onto the WS_2_ flakes prepared atop a sapphire substrate. After 5 min at 80 °C, a water droplet was placed on top of the PMMA layer and the PMMA edge was poked with a sharp tweezer to allow water to penetrate underneath the WS_2_ layer; this resulted in WS_2_-sapphire separation. The resulting PMMA-WS_2_ film was then transferred onto the TSG and heated for 10 min at 80 °C to remove water. The PMMA was then dissolved away by using acetone.

### 2.5. Raman, AFM, and PL Instrumentation

All Raman and PL measurements were performed by using a LabRam HR (Horiba, Edison, NJ, USA) with a 100× objective (LM Plan FL N, NA = 0.80, Olympus, Center Valley, PA, USA). The excitation source was a 532.06 nm laser (Laser Quantum, Fremont, CA, USA). Detection was performed by using a one-electrode thermoelectrically cooled CCD detector (Andor Technology Inc., Belfast, Northern Ireland). The entire system was controlled by LabSpec 6 software (Horiba).

Atomic force microscopy (AFM) measurements were recorded by using an AIST-NT model SmartSPM 1000 in intermittent contact mode with an Al-coated Si probe (k = 2 N/m, 240 µm length) (AC240TM-R3, Oxford Instruments Asylum Research, Santa Barbara, CA, USA) operating at a 70 kHz resonant frequency and controlled by Omega software 3.5.81 (AIST-NT). 

### 2.6. AFM and Raman Studies

Atomic force microscopy height and phase images were recorded from 10 µm × 10 µm areas at a 512 × 512-pixel resolution (ca., 19.5 nm/step). Co-localized Raman and PL emission maps were acquired from 10 µm × 10 µm areas at a 64 × 64-pixel resolution (ca., 156 nm/step). All Raman and PL experiments were performed by using 600 and 300 grooves/mm gratings, respectively, yielding 1.6 cm^−1^ and 3.1 nm spectral resolutions, respectively. The incident laser power density for all Raman and PL experiments was 510 W/cm^2^ and 130 W/cm^2^, respectively. All image and maps were recorded at 1.0 Hz/pixel.

### 2.7. Gaseous Analyte Vapor Studies

The WS_2_ specimens were placed within a custom-built Teflon flow cell with a glass optical port. The flow cell was maintained at room temperature (294–296 K) while the WS_2_ specimen was subjected to air or acetone vapor environments. The air/acetone cycle was performed at least three times on the same specimen to determine reproducibility (no detectable hysteresis). At least three specimens were assessed. Typical results are shown.

## 3. Results and Discussion

### 3.1. Layer Thickness Determination

**Figure 1:**
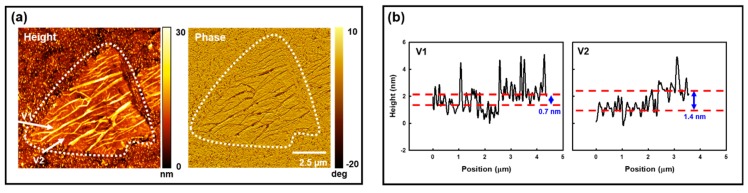
Typical AFM results for a WS_2_ flake on TSG. (**a**) Typical AFM height (**left**) and phase (**right**) images. The dotted curve is used to outline the WS_2_ flake. Two vectors (V1 and V2) are shown in the height image. (**b**) Typical height profiles along the V1 (**left**) and V2 (**right**) vectors shown in panel a. The V1 and V2 vectors traverse largely single- and two-layer regions, respectively.

Figure 1a presents typical AFM height (left) and phase (right) images from a WS_2_ flake on TSG. The WS_2_ appears to be wrinkled and ridges in the height image correspond to areas with low phase values (e.g., −20°) whereas regions that appear less wrinkled have more uniform, higher phase values (e.g., +10°). Phase imaging in AFM is a measure of the energy dissipation between the AFM probe tip and the sample. This depends on many factors, including specimen-to-substrate adhesion [23] (pp. 69–71). In our case, a lower phase value indicates lower adhesion between regions within the WS_2_ flake and the Au substrate while higher phase values indicate greater adhesion between regions within the WS_2_ flake area and the Au substrate. The results shown in Figure 1a are consistent with WS_2_ flakes that are wrinkled on the TSG surface where the WS_2_ ridges are in poor contact with the Au substrate.

In Figure 1b, we present AFM height profiles along the two vectors shown in Figure 1a (left). The V1 vector traverses a pathway that is largely single-layer WS_2_ and the V2 vector follows a path that appears to be largely composed of two-layer WS_2_. Unfortunately, the WS_2_ wrinkles make it very challenging to assess our WS_2_ flake thickness by using AFM alone. 

**Figure 2:**
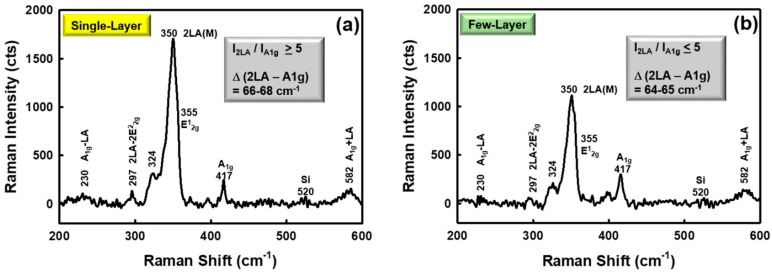
Typical single point Raman spectra and band assignments for different areas on a WS_2_ flake on TSG. (**a**) Single-layer region. (**b**) Few-layer region.

Figure 2 presents typical diffraction limited, single-point Raman spectra recorded at WS_2_ flake edges devoid of wrinkles that were determined by AFM height measurements to be either single- (Figure 2a) or few-layer (Figure 2b). All detectable Raman bands are labeled [2], including the key 2LA, E^1^_2g_, and A_1g_ bands. Using the known AFM-derived height at specific locations across a WS_2_ flake, the boxed insets in Figure 2a,b show that the *I_2LA_*/*I_A1g_* and *Δ* (difference in the 2LA-A_1g_ band maxima) are layer number dependent and can be readily used to distinguish single- and few-layer WS_2_ even on a heavily wrinkled specimen.

**Figure 3:**
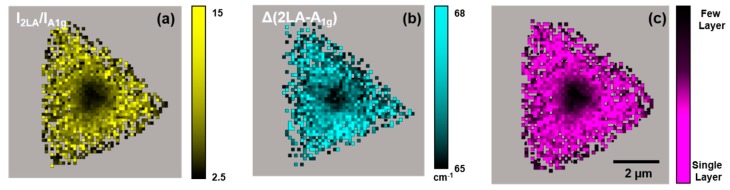
Typical WS_2_ layer count maps for a single WS_2_ flake on TSG. (**a**) *I_2LA_*/*I_A1g_* intensity ratio map. (**b**) *Δ* (E_2LA_ − E_A1g_) band energy difference map. (**c**) Combined a and b WS_2_ layer count map.

Figure 3 presents typical Raman-based mapping results from an isolated WS_2_ flake on TSG. Figure 3a,b illustrate the *I_2LA_/I_A1g_*- and *Δ*-based maps, respectively (the original 2LA and A_1g_ intensity and position maps can be found in the Supplementary Materials in Figures S1 and S2). Figure 3c presents a layer count map created by multiplying Figure 3a,b. An inspection of these data shows that this particular WS_2_ flake has a center region that consists of few-layer (mostly two-layer) WS_2_ and the flake remainder is composed, largely, of single-layer WS_2_.

### 3.2. Acetone Effect on WS_2_ Flake Total PL and Exciton Band Emission

**Figure 4:**
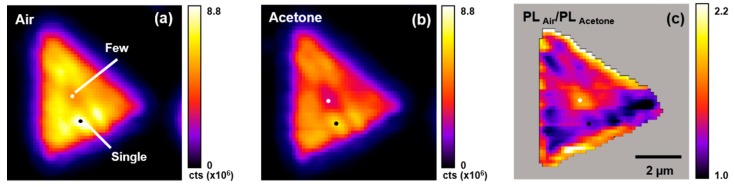
Typical total PL emission intensity maps (1.8–2.1 eV) for a single WS_2_ flake on TSG. (**a**) Air atmosphere. (**b**) Acetone atmosphere. (**c**) *PL_Air_*/*PL_Acetone_* intensity response map. The black and white dots indicate single- and few-layer WS_2_ regions determined from Figure 3c.

Figure 4 presents typical total PL emission intensity (1.8–2.1 eV) maps for the WS_2_ flake shown in Figure 3 under air (Figure 4a) or acetone vapor atmospheres (Figure 4b). Two specific locations on this WS_2_ flake are labeled in Figure 4a, denoting regions that are composed of single- (black circle) and few-layer (white circle) WS_2_. These locations were selected based on the material classification scheme shown in Figure 3. Figure 4c presents the PL intensity ratio (*PL_Air_*/*PL_Acetone_*) map from the data in Figure 4a,b. An inspection of these data shows that the PL emission intensity: (i) Is heterogeneous across the WS_2_ flake when it is under air or acetone vapor, (ii) is generally quenched by acetone vapor, and (iii) quenching is heterogeneous across the WS_2_ flake. The largest extent of PL quenching is observed at the flake’s middle and upper and lower edges, where the flake is mostly composed of few-layer WS_2_ (cf., Figure 3). Thus, there is a strong spatial dependence in the acetone-induced PL emission quenching from a WS_2_ flake.

**Figure 5:**
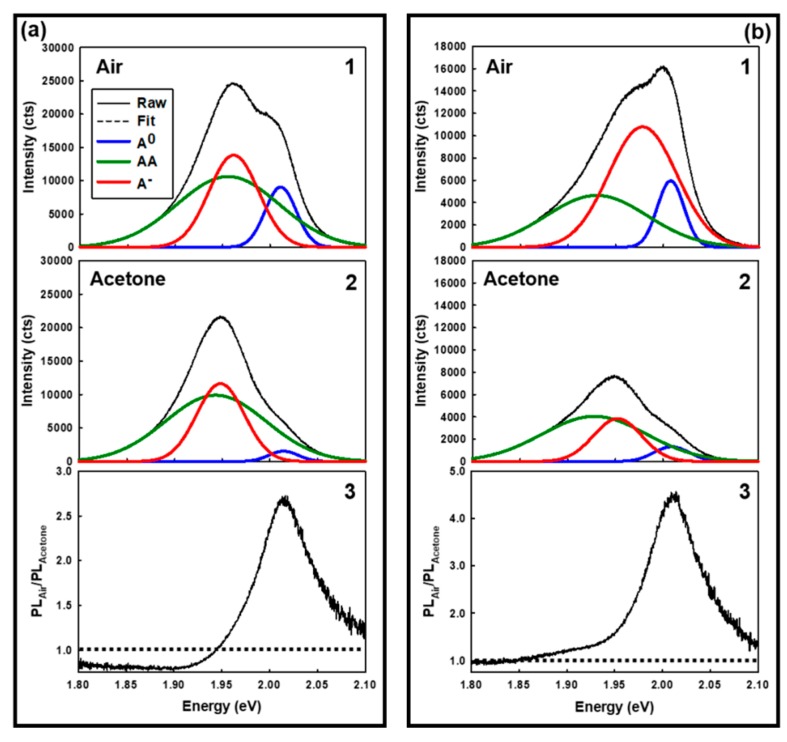
Typical single point PL emission spectra and curve fits from specific locations on the WS_2_ flake shown in Figure 4. (**a**) Spectra from a single-layer WS_2_ region. (**b**) Spectra from a few-layer WS_2_ region. (1) Spectra recorded in air. (2) Spectra recorded under acetone vapor. (3) Energy-dependent *PL_Air_*/*PL_Acetone_* response spectra. The dashed line in spectra 3 located at 1.0 denotes no quenching. Spectral regions with *PL_Air_*/*PL_Acetone_* < 1 exhibit a PL enhancement under acetone vapor. Spectral regions with *PL_Air_*/*PL_Acetone_* > 1 exhibit a PL quench under acetone vapor.

Figure 5 present typical diffraction limited, single-point PL emission spectra from the WS_2_ specimen shown in Figure 3 measured at locations within the flake (cf., Figure 3c) that are composed of single- (Figure 5a) or few-layer (Figure 5b) WS_2_. Within each panel series are spectra recorded in air (1) and under acetone vapor (2) plus the corresponding PL emission energy-dependent *PL_Air_*/*PL_Acetone_* response spectra (3). The dashed line in spectra 3 (located at 1.0) denotes where there is no detectable PL emission quenching. Energy regions with *PL_Air_*/*PL_Acetone_* values < 1 exhibit acetone-induced PL enhancement whereas energy regions with *PL_Air_*/*PL_Acetone_* values > 1 exhibit acetone-induced PL quenching. The PL emission spectra labeled 1 and 2 were individually curve fitted to models having two or three exciton emission bands (Gaussian). The best fit (r^2^ > 0.99) was observed for a three band model and is consistent with emission from the A^0^, AA, and A^−^ excitons as reported by other researchers [4,18,24]. The general assignment of each WS_2_ exciton band in the current research is based on previous reports in the literature [4,16,18,24]. We have also noticed that the AA exciton band exhibits a laser power-dependent position shift and a non-linear increase in amplitude with increasing laser power (results not shown). This behavior is consistent with an AA exciton being present [16]. Together, these data show that there are dramatic differences in the A^0^, AA, and A^−^ exciton emission susceptibility and response to acetone vapors and also a strong WS_2_ layer number dependency. This has significant ramifications in the construction of PL-based sensors using such a platform and illustrates several potential opportunities for the creation of a tailored sensor response. For example, in quenchometric sensors [25,26], the quencher (Q) causes the PL emission intensity (*PL*) to decrease in a Q-dependent manner [25,26]. A response curve is created by plotting *PL_0_*/*PL_analyte_* vs. *[Q]*, where *PL_0_* and *PL_analyte_* represent the PL emission intensities in the absence and presence of Q, respectively. An ideal quenchometric sensor exhibits a response curve with high sensitivity (i.e., d((*PL_0_*/*PL_analyte_)/*d*[Q]*) [25,26]. 

**Figure 6:**
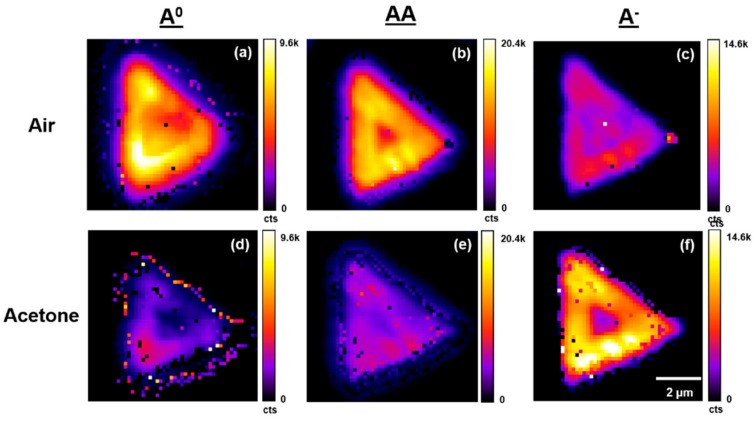
Typical PL emission A^0^, AA, and A^−^ exciton band amplitude maps for a single WS_2_ flake on TSG under air and acetone vapors. Maps are generated by curve fitting PL emission spectra at each pixel across the entire flake. (**a**,**b**,**c**) Maps in air. (**d**,**e**,**f**) Maps under acetone vapor.

**Figure 7:**
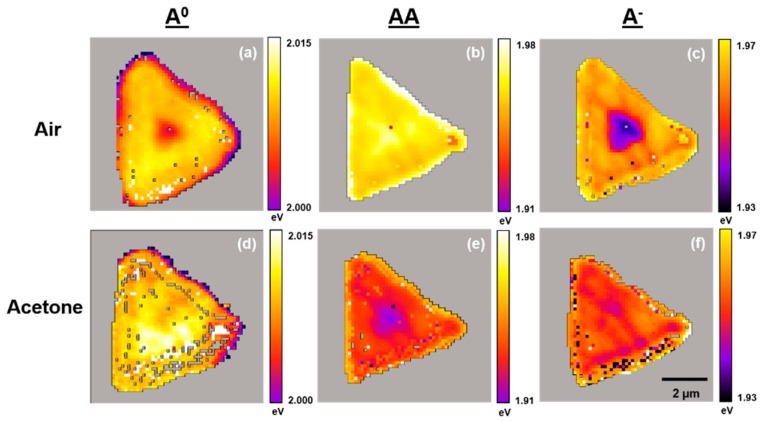
Typical PL emission A^0^, AA, and A^−^ exciton band maxima (eV) maps for a single WS_2_ flake on TSG under air and acetone vapors. Maps are generated by curve fitting PL emission spectra at each pixel across the entire flake. (**a**,**b**,**c**) Maps in air. (**d**,**e**,**f**) Maps under acetone vapor.

**Figure 8:**
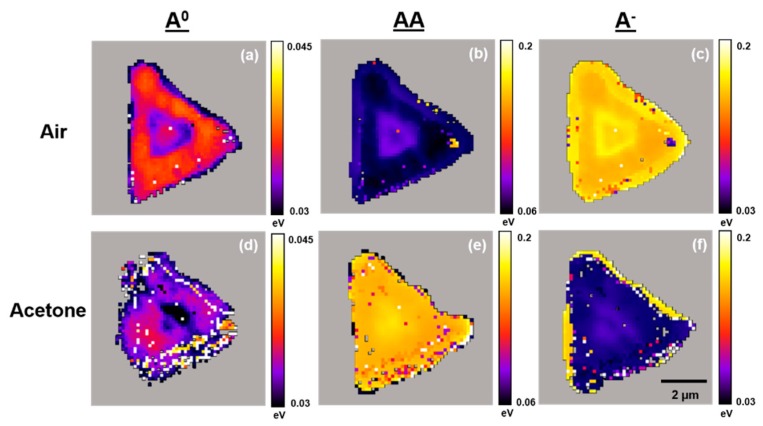
Typical PL emission A^0^, AA, and A^−^ exciton band FWHM (eV) maps for a WS_2_ flake on TSG under air and acetone vapors. Maps are generated by curve fitting PL emission spectra at each pixel across the entire flake. (**a**,**b**,**c**) Maps in air. (**d**,**e**,**f**) Maps under acetone vapor.

Figure 6, Figure 7 and Figure 8 present typical A^0^, AA, and A^−^ PL emission exciton band amplitude, band maxima (eV), and band full width at half maximum (FWHM) (eV) maps, respectively, for the WS_2_ flake shown in Figure 3 and Figure 4. These air- and acetone-dependent exciton species maps were created by curve fitting the individual PL emission spectra in air and under acetone vapor at each pixel within the region of interest (4096 separate spectra) as described in Figure 5. 

**Figure 9:**
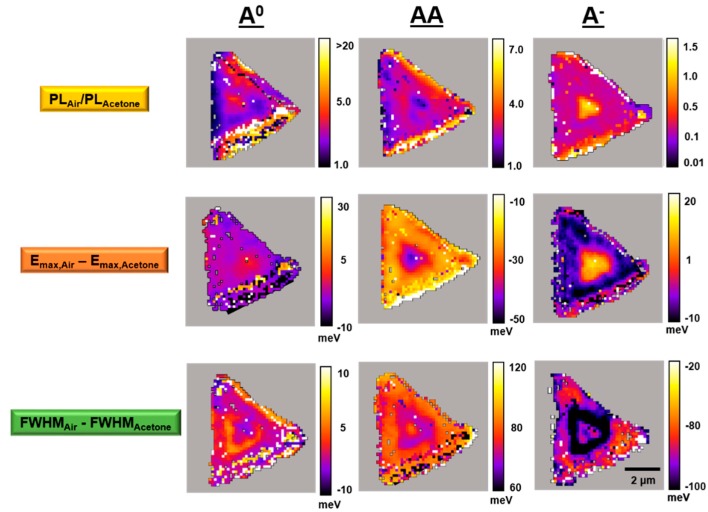
Typical acetone vapor-dependent PL emission A^0^, AA, and A^−^ exciton band intensity ratio (*PL_Air_*/*PL_Acetone_*), band energy maxima difference (*E_max,Air_* − *E_max,Acetone_*), and band FWHM energy difference (*FWHM_Air_* − *FWHM_Acetone_*) maps for a single WS_2_ flake on TSG.

Figure 9 summarizes the impact of acetone vapor on the A^0^, AA, and A^−^ exciton emission bands from an individual WS_2_ flake on TSG. Several aspects of these results merit further discussion. In a general sense, the response depends on the emission exciton type and WS_2_ layer number. The A^0^ exciton from single-layer WS_2_ exhibits the highest acetone-induced quenching sensitivity (*PL_Air_*/*PL_Acetone_* up to 20). The A^0^ exciton from few-layer WS_2_ exhibits an acetone-induced quenching sensitivity that is generally 1/4 the corresponding single-layer value. The AA exciton from single-layer WS_2_ is only 1/3 as sensitive (*PL_Air_*/*PL_Acetone_* up to 7) to acetone-induced quenching in comparison to the single-layer A^0^ exciton. The AA exciton from few-layer WS_2_ is not particularly sensitive to acetone-induced quenching (1.0 > *PL_Air_*/*PL_Acetone_* < 1.5). The A^−^ exciton exhibits unique PL emission behaviors. The A^−^ exciton from few-layer WS_2_ exhibits only modest acetone-induced quenching sensitivity (1.1 > *PL_Air_*/*PL_Acetone_* < 1.5). Interestingly, the A^−^ exciton band from the lion’s share of single-layer WS_2_ is the only band within the flake to exhibit an acetone-induced PL emission enhancement (0.1 > *PL_Air_/PL_Acetone_* < 0.5). It is well known that acetone acts as an electron donor in the presence of MoS_2_ and WS_2_ [9,15,27,28]. A previous study on MoS_2_ [15] shows that the positive trion (one electron, two holes, A^+^) band amplitude decreases in the presence of an electron donor, and the authors reasoned that when additional electrons are introduced from an electron donor, the PL spectrum is suppressed because of dissociation of the positive trions from the neutral excitons (A^0^). We determine the enhancement seen in our results is from the negative trion (two electrons, one hole, A^−^) accepting additional electrons from the electron donor, which results in an increase in the PL spectrum for the A^−^.

Acetone also induces detectable shifts in the exciton emission band energies. The energy shifts in the A^0^ exciton emission band across the WS_2_ flake are the least distinct, but there are up to +30 meV shifts from regions that also exhibit the highest acetone susceptibilities (single-layer WS_2_). The energy shifts within the few-layer region are very small (just +5 meV) for the A^0^ exciton emission band. The energy shifts in the AA exciton emission band across the WS_2_ flake produces a very distinct pattern. In the single-layer region, the acetone-induced shift is up to −10 meV. In the few-layer region, the corresponding shift is up to −50 meV. This could arise from the topmost WS_2_ layer within the few-layer region. The energy shifts in the A^−^ exciton band emission across the WS_2_ flake produces another distinct pattern. In the single-layer region, the acetone-induced shift is small (0 to +10 meV). In the few-layer region, the corresponding shift is up to +20 meV.

The exciton band FWHM also changes under acetone vapor. The FWHM shifts in the A^0^ exciton emission band across the WS_2_ flake show very modest effects (−10 to +10 meV), but the more positive shifts appear in areas with large energy differences and the highest sensitivity to acetone quenching. The FWHM shifts in the AA exciton emission band across the WS_2_ flake exhibit an interesting pattern. In single-layer WS_2_ regions with high AA exciton emission band sensitivity to acetone, the band FWHM shift is up to +60 meV. In the few-layer WS_2_ region, the band FWHM shifts are slightly larger (up to +80 meV). This could arise from the topmost WS_2_ layer within the few-layer region. The exciton emission band FWHM shifts in the A^−^ exciton band across the WS_2_ flake exhibit a striking pattern. In the single-layer region, the band FWHM shifts are generally −50 to −80 meV, but there are regions with −20 meV shifts at the flake edges that also correspond to a high PL quenching sensitivity. In the immediate area surrounding the few-layer region, the FWHM shifts are the largest (up to −100 meV). This area corresponds to the region where acetone-induced PL enhancement is observed.

**Figure 10:**
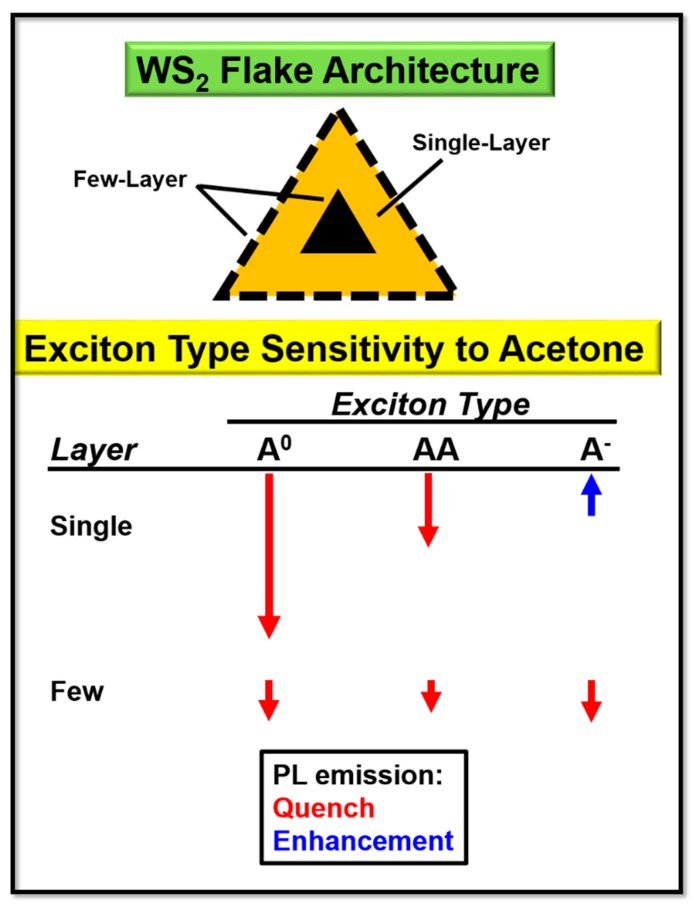
Model of the overall effect of acetone on exciton bands. A WS_2_ flake in an air environment denoting single- and few-layer areas. The red arrows indicate a quench in PL and the blue arrow indicates an enhancement. The arrow length represents the effect magnitude.

Figure 10 is a model to summarize the single- and few-layer WS_2_ exciton PL emission behavior in the presence of acetone. Acetone causes an overall decrease in the A^0^ and AA exciton band PL emission for single- and few-layer WS_2_. A decrease in PL emission was also produced in the A^−^ exciton band PL emission in few-layer WS_2_, but there is an overall increase in the A^−^ exciton band PL emission from single-layer WS_2_.

## 4. Conclusions

Atomic force microscopy, Raman, and PL maps were used to determine the WS_2_ layer thickness and map the A^0^, AA, and A^−^ PL emission exciton band contribution within the flake for chemical sensing assessment. Analyte vapor studies show that the PL response from the WS_2_ flake is exciton-type and layer number dependent. It has been shown that the exciton amplitude, energy, and FWHM are all affected by acetone vapor. The A^0^ exciton PL emission is most strongly quenched by acetone whereas the A^−^ exciton PL emission shows a unique PL enhancement under acetone vapors. The results identify interesting strategies to generate unique, analyte-dependent responses from single- and few layer WS_2_ platforms. We are currently investigating single- and few-layer WS_2_ PL exciton band behavior on sapphire and SiO_2_/Si substrates.

## Supplementary Materials

The following are available online at https://www.mdpi.com/1424-8220/19/8/1913/s1, Figure S1: Initial Raman maps and layer count ratio. Figure S2: Initial Raman maps and layer count difference. Figure S3: Simplified schematic depicting WS_2_ fabrication.
