# Supplementary material for: Effects of Acetone Vapor on the Exciton Band Photoluminescence Emission from Single- and Few-Layer WS2 on Template-Stripped Gold
Source: Sensors (Basel). 2019 Apr 23;19(8):1913. doi: 10.3390/s19081913 (PMC6514759; doi:10.3390/s19081913)
Supplement: Supplementary file 1 [file sensors-19-01913-s001.pdf]

sensors

MDPI

Supplementary Materials

Effects of Acetone Vapor on the Exciton Band Photoluminescence Emission from Single- and Few-Layer WS_2 on Template-Stripped Gold

Samantha Matthews, Chuan Zhao, Hao Zeng and Frank V. Bright

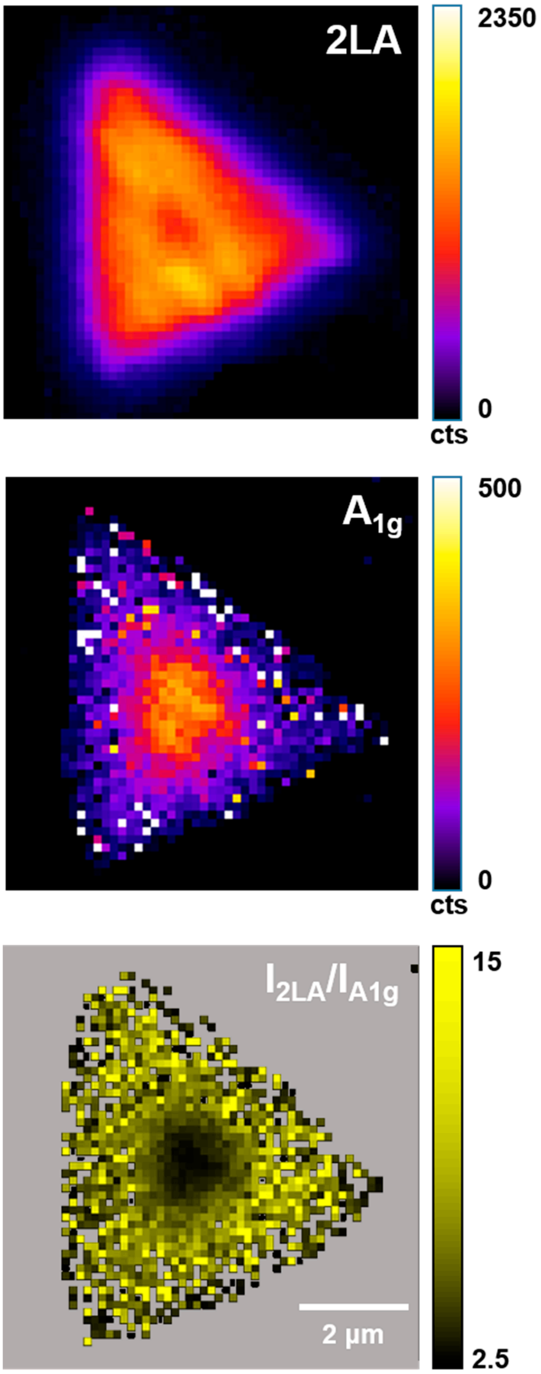

Figure S1. Initial Raman maps and layer count ratio.

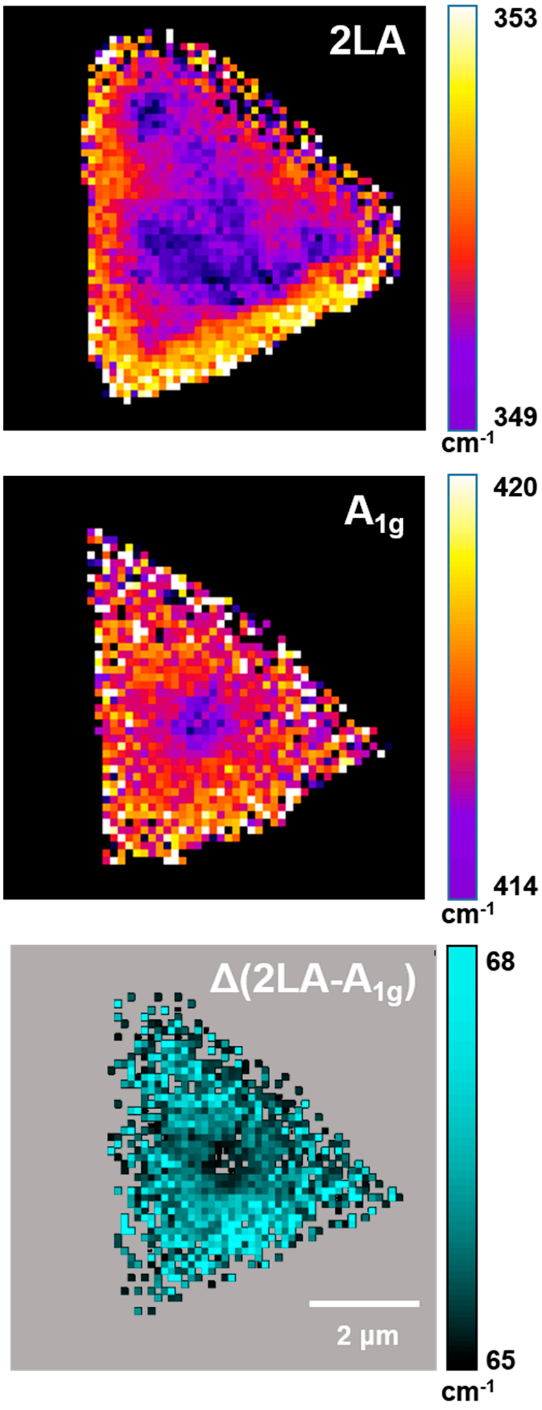

Figure S2. Initial Raman maps and layer count difference.

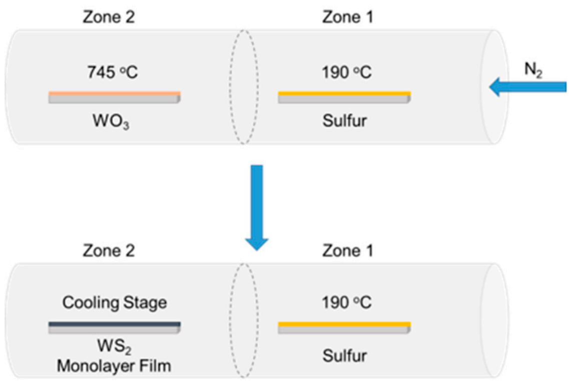

Figure S3. Simplified schematic depicting WS_2 fabrication.
